# Evaluating the Implementation of Integrated Proactive Supportive Care Pathways in Oncology: Master Protocol for a Cohort Study

**DOI:** 10.2196/52841

**Published:** 2024-08-26

**Authors:** Maria Alice Franzoi, Arnaud Pages, Loula Papageorgiou, Antonio Di Meglio, Ariane Laparra, Elise Martin, Aude Barbier, Nathalie Renvoise, Johanna Arvis, Florian Scotte, Ines Vaz-Luis

**Affiliations:** 1 Cancer Survivorship Group (INSERM U981) Gustave Roussy Villejuif France; 2 Department of Biostatistics and Epidemiology Gustave Roussy Villejuif France; 3 Interdisciplinary Department for the Organization of Patient Pathways – DIOPP Gustave Roussy Villejuif France

**Keywords:** care delivery, pathway of care, oncology, supportive care, quality of life, cohort study

## Abstract

**Background:**

Supportive care (SC) refers to the prevention and management of complications of cancer and its treatment. While it has long been recognized as an important cancer care delivery component, a high proportion of patients face unaddressed SC needs, calling for innovative approaches to deliver SC.

**Objective:**

The objective of this master protocol is to evaluate the implementation of different integrated proactive SC pathways across the cancer care continuum in our institution (Gustave Roussy, Villejuif, France). Pathways studied in this master protocol may occur shortly after diagnosis to prevent treatment-related burden; during treatment to monitor the onset of toxicities and provide timely symptom management; and after treatment to improve rehabilitation, self-management skills, and social reintegration.

**Methods:**

This study is guided by the Reach, Effectiveness, Adoption, Implementation, and Maintenance framework. The primary objective is to evaluate the impact of SC pathways on patients’ distress and unmet needs after 12 weeks, measured by the National Comprehensive Cancer Network’s Distress Thermometer and Problem List. Secondary objectives will focus on the pathways (macrolevel) and each SC intervention (microlevel), evaluating their reach (administrative data review of the absolute number and proportion of clinical and sociodemographic characteristics of patients included in the pathways); short-term and long-term efficacy through their impact on quality of life (EQ-5D-5L and the 30-item European Organization for Research and Treatment of Cancer Quality of Life Core Questionnaire) and symptom burden (MD Anderson Symptom Inventory, Hospital Anxiety and Depression Scale, Insomnia Severity Index, and 22-item European Organization for Research and Treatment of Cancer Sexual Health Questionnaire); adoption by patients and providers (administrative data review of SC referrals and attendance or use of SC strategies); barriers to and leverage for implementation (surveys and focus groups with patients, providers, and the hospital organization); and maintenance (cost-consequence analysis). Pilot evaluations with a minimum of 70 patients per pathway will be performed to generate mean Distress Thermometer scores and SDs informing the calculation of formal sample size needed for efficacy evaluation (cohorts will be enriched accordingly).

**Results:**

The study was approved by the ethics committee, and as of February 2024, a total of 12 patients were enrolled.

**Conclusions:**

This study will contribute toward innovative models of SC delivery and will inform the implementation of integrated SC pathways of care.

**Trial Registration:**

ClinicalTrials.gov NCT06479057; https://clinicaltrials.gov/study/NCT06479057

**International Registered Report Identifier (IRRID):**

PRR1-10.2196/52841

## Introduction

### Background

Due to the rapid evolution of modern anticancer treatment, cancer is often considered a chronic disease. Novel treatments deliver longer survival but frequently with a range of acute toxicities and long-term side effects, which negatively impact the quality of life and necessitate ongoing health service use [1].

Supportive care (SC) is defined as the prevention and management of adverse effects of cancer and its treatment [2,3]. This includes management of physical and psychological symptoms and side effects across the continuum of cancer experience, from diagnosis through anticancer treatment to posttreatment care [2,3]. Enhancing rehabilitation, secondary cancer prevention, survivorship, and end-of-life care are integral to SC. Financial and social issues that may be associated with risk of toxicities or access to SC should also be considered [2,4-6].

While SC has long been recognized as an important component of cancer care delivery, published evidence suggests an ongoing, high burden of unaddressed needs across all SC domains for many patients, at all phases of cancer experience [7,8]. A systematic review of 57 studies quantifying patients’ unmet SC needs across different tumor types and phases of the cancer continuum reported that, although highly variable, these could reach 93% for unmet informational needs, 89% for unmet physical needs, 89% for unmet psychosocial needs, 63% for unmet sexuality needs, and 51% for unmet spiritual needs, with many of these unmet needs experienced concurrently [9]. In addition, while unmet needs appeared to be highest and most varied during treatment, a greater number of individuals were likely to express unmet needs after the end of treatment compared to any other time [9]. More specifically, long-term physical and behavioral symptoms (fatigue, neuropathy, weight management, emotional distress, insomnia, and cognition decline), intimacy-related concerns, financial and work-related concerns, and provider-communication and information needs have been highly cited as common unmet needs across the cancer care continuum [10].

A recent study aimed to identify and synthesize patient’s views about areas where they need support through cancer care [8]. Quantitative and qualitative studies were included, and the authors presented 3 lines of work for managing chronic illness (“illness-work,” “everyday-life work”, and “biographical work”), including a group of key common patient needs. For “illness-work,” the key needs identified were understanding their illness and treatment options, knowing what to expect, communication with health care professionals, and staying well. With regard to “everyday-life work”, patients wanted to maintain a sense of normalcy and look after their loved ones. For “biographical work,” patients commonly struggled with the emotional impact of illness and a lack of control over their lives. Spiritual, sexual, and financial problems were less universal. For some types of support, demographic factors influenced the level of need reported.

Importantly, there is evidence demonstrating that unmet SC needs are associated with inferior quality of life, increased symptom burden, and worse clinical outcomes such as emergency visits and hospitalizations [11-13].

Although a large body of evidence exists on interventions addressing SC needs in patients with cancer [14], implementation of effective interventions in clinical practice has been suboptimal [7,15]. The delivery of SC requires a multidisciplinary approach, involving the screening, assessment, management, treatment of side effects, symptoms, and needs of patients with cancer, carriers, and family [2]. A recent framework highlighted the importance of perceiving SC as a way of understanding, planning, delivering, and evaluating integrated cancer care, where each component of cancer care and treatment occur within a SC framework, instead of considering it as a subspecialty, discipline, or series of interventions [16]. Ideally, SC needs to be delivered in all health care settings at all steps of the cancer pathway from diagnosis to survivorship and end of life [2]. Most commonly, SC is primarily delivered in everyday practice by treating oncologists who during their routine oncological consultation may detect SC needs and refer patients to multiple multidisciplinary strategies such as palliative care, social work, rehabilitation, psycho-oncology, and integrative medicine. However, this approach can lead to heterogeneous access and fragmented care [16]. An integrated SC delivery model has been proposed to overcome these challenges [17]. In this model, longitudinal specialist SC is provided by an interdisciplinary SC team, with timely involvement of other teams (eg, cancer pain service and rehabilitation) when the need arises [17]. For patients who have completed curative treatments and are on surveillance, the survivorship care team may be the main SC service [18]. Key features of this model include universal referral, systematic SC needs screening, tailored specialist involvement and evidence-based symptom management strategies, streamlined care, and collaborative teamwork [17]. In addition, several modifiable risk factors for toxicity and quality of life deterioration have been identified across the cancer care continuum [19-23]. Therefore, an integrated preventive strategy that anticipates tailored SC services to patients at higher risk of toxicity or quality of life deterioration has been suggested [22] and requires further evaluation of its implementation and impact on patient-reported outcomes.

### Objectives

**Figure 1:**
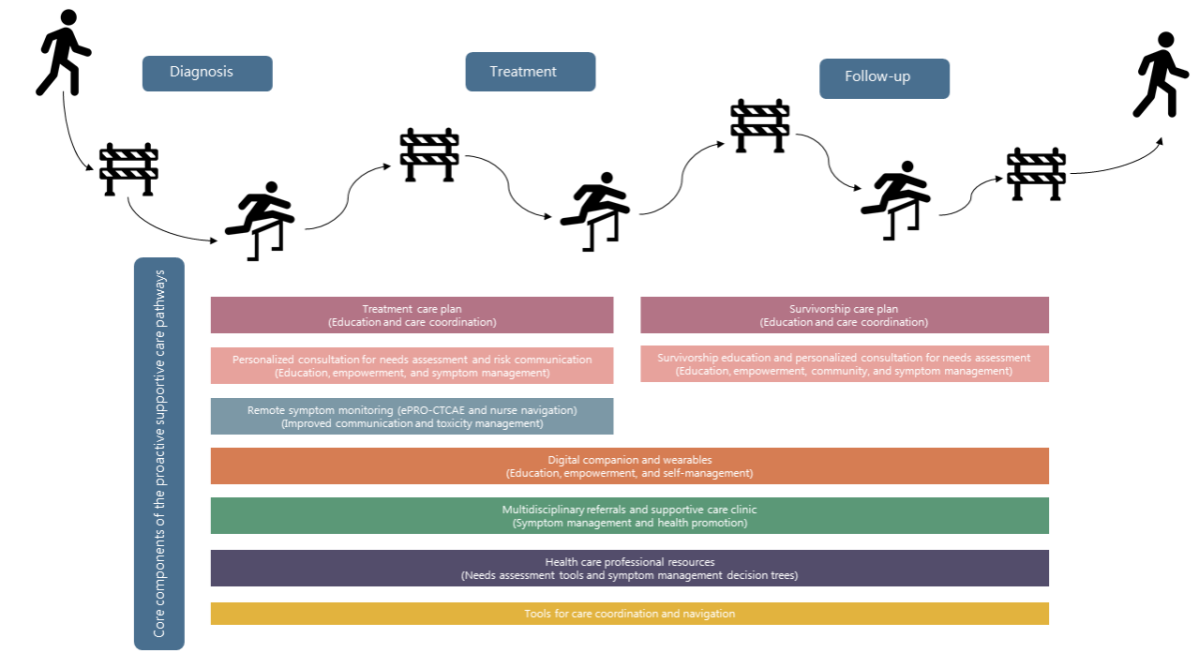
Examples of core supportive care components of supportive care pathways across the cancer care continuum. ePRO-CTCAE: electronic patient-reported outcomes version of the common terminology criteria for adverse events.

At our institution (Gustave Roussy, Villejuif, France), several SC resources were co-designed and deployed in clinical practice (Figure 1 and Textbox 1), including integrated SC pathways of care and specific SC referrals. In Gustave Roussy, the integrated SC pathways include a formal SC needs assessment and tailored SC referrals and resources according to patient’s needs. These pathways are offered to patients across the entire cancer care continuum: shortly after diagnosis to prevent treatment-related burden; during treatment to monitor and manage treatment-related toxicities; and after treatment to accelerate rehabilitation, social integration, and self-management. This study will evaluate the implementation of integrated and proactive SC pathways in oncology.

**Current supportive care interventions and referrals available at Gustave Roussy.**

**Integrated supportive care pathways**

- Shortly after diagnosis
  Supportive care pathway for preventing treatment-related burden after breast cancer (eg, cancer-related fatigue and cluster of behavioral symptoms)
  Supportive care pathway to prevent treatment-related burden for patients considered vulnerable with brain, head and neck, thoracic, and neuroendocrine tumors
- During treatment
  Remote symptom monitoring pathway with weekly electronic patient-reported outcomes (ePROs) and nurse navigation (for patients during active systemic treatment with chemotherapy, immunotherapy, or targeted agents)
  Immunotoxicity management pathway
- After treatment
  Proactive survivorship care pathway (breast cancer and others)

**Supportive care interventions available at the institution**

- Digital health tools
  Remote monitoring (ePRO system) for patients receiving active treatment (chemotherapy, target agents, and radiotherapy)
  Mobile app delivering education and self-management advice (articles, videos, and podcasts)
  Internet-delivered self-care programs of evidence-based validated supportive care strategies (physical activity, mindfulness, yoga, and cognitive behavioral therapy)
  ePRO data collection web system for research studies
- In-person multidisciplinary supportive care resources at the supportive care clinic
  Adapted physical activity program
  Mindfulness program
  Acupuncture consultations
  Hypnosis consultations
  Sophrology program
  Art-therapy program
  Socioesthetician follow-up
  Return-to-work educational seminars
  Diet and nutrition educational seminars
  Survivorship educational seminars
  Individualized risk of toxicity assessment
- Community-based survivorship resources
  Nutrition follow-up
  Adaptive physical activity
  Psychological support
- In-person multidisciplinary supportive care resources at the hospital
  Nutritionist follow-up
  Palliative care follow-up
  Addiction treatment programs (tobacco and alcohol)
  Social services follow-up
  Organ specialists for toxicity management of cancer therapies (cardiologist, pneumologist, endocrinologist, neurologist, rheumatologist, etc)
  Day hospital for acute toxicity management
  Rehabilitation hospital
  Psychologist or psychiatrist follow-up
  Pain evaluation and management at pain clinic
  Sexologist follow-up
  Speech therapy
  Ostomy nurses
  Supportive care nurses
  Thrombosis evaluation team
  Bone metastases evaluation team

## Methods

### Study Design and Interventions

The study design is a master protocol [24] for a prospective cohort study focused on evaluating the implementation of integrated proactive pathways of SC at Gustave Roussy. This master protocol study is conducted with a collection of substudies for each SC intervention that share key design components and operational aspects. Data collection will allow the evaluation at a macro level (integrated SC pathway, including SC needs assessment and tailored multidisciplinary referrals) and at a micro level (separated for each SC intervention).

The Reach, Effectiveness, Adoption, Implementation, and Maintenance (RE-AIM) framework [25] was used to guide the definition of study objectives and the data collection plan. As a framework, RE-AIM has both individual-level and staff- or setting-level dimensions, including Reach and Effectiveness (individual-level), Adoption and Implementation (staff and setting levels), and Maintenance (both individual and staff or setting levels) [26].

Patients will be clinically monitored from the date of their screening visit until the date of the last visit, loss to follow-up, withdrawal of consent, disease progression, death, or end of study, whichever occurs first. There are no treatments prescribed to the patients other than those prescribed in routine practice.

Patients will be prospectively entered in the study after checking for eligibility criteria and signature of the informed consent form. Data will be collected at baseline, 4 weeks, 12 weeks, and 24 weeks. For patients participating in the immunotoxicity management pathway, data will also be collected at 1 month. Study participants will be asked to complete the study web-based questionnaires using the WeShare platform’s electronic patient-reported outcomes module [27,28]. Identifying data (personal data) are recovered at the time of the creation of the WeShare account and will be separated from the study data. The investigation plan is detailed in Table 1. In-paper questionnaires will also be available if preferable by the patient.

**Table 1:** Investigation schedule.

		Electronic case report form creation	Baseline visit	Follow-up month 1^a^ week 4 (–2 weeks to +2 weeks)	Follow-up month 3 week 12 (–2 weeks to +2 weeks)	Follow-up month 6 week 24 (–2 weeks to +2 weeks)
Prescreening informed consent		✓				
Screening informed consent			✓			
Eligibility criteria			✓			
Clinical data^b^			✓	✓	✓	✓
Supportive care resources proposed^c^			✓			
Attendance and adoption data to supportive care resources proposed^d^					✓	✓
Out of schedule use of hospital services (emergency visits, extra consultations, and hospitalization)					✓	
Resilience mobile app use data^e^			✓	✓	✓	✓
**Electronic patient-reported outcomes^f^**						
	Sociodemographic questionnaire		✓			
	National Comprehensive Cancer Network’s Distress Thermometer and Problem List		✓	✓	✓	✓
	5-level EQ-5D version		✓	✓	✓	✓
	MD Anderson Symptom Inventory		✓	✓	✓	✓
	Patient Assessment of Chronic Illness Care				✓	
	Health Literacy Questionnaire		✓			
	Gustave Roussy vulnerability questionnaire^g^		✓			
	30-item European Organization for Research and Treatment of Cancer Quality of Life Core Questionnaire^h^		✓		✓	✓
	Hospital Anxiety and Depression Scale^i^		✓		✓	✓
	Insomnia Severity Index ^j^		✓		✓	✓
	22-item European Organization for the Research and Treatment of Cancer Sexual Health Questionnaire^k^		✓		✓	✓
	Experience and satisfaction questionnaires^l^				✓	
**Qualitative research**						
	Focus groups^m^				✓	

^a^For patients included in the immune toxicity management pathway.

^b^Complete clinical data including type of cancer; stage; prior and ongoing oncological treatments; comorbidities; disease status; description and grading of treatment-related toxicities and its relationship with treatment; treatment discontinuation and reintroduction; supportive care strategies proposed and attendance log to supportive care strategies; and use of hospital services, including emergency visits, extra consultations, and hospitalizations. Oncological scores used for supportive care management included: Geriatric score G8, thrombosis score, nausea, and vomiting score.

^c^For the complete list of supportive care resources, refer to Textbox 1.

^d^Including attendance data in all in-person supportive care interventions.

^e^Resilience [29] is a mobile app used in routine care at Gustave Roussy for remote symptom monitoring, patient empowerment, and education. Data on mobile app use, including symptoms reported, alerts generated, and content used, will be evaluated.

^f^Specific questionnaires can be added if pertinent to a new pathway.

^g^Sent only for patients participating in the pathway to prevent treatment-related burden (brain, head and neck, thoracic, and neuroendocrine tumors).

^h^Sent only for patients in the pathway for preventing treatment-related burden after breast cancer.

^i^Sent only for patients in the pathway for preventing treatment-related burden after breast cancer or patients referred to supportive care services for emotional distress and anxiety (mindfulness meditation programs, cognitive behavioral therapy, and psychological consultation).

^j^Sent only for patients referred for supportive care strategies for insomnia (cognitive behavioral therapy and mindfulness meditation programs).

^k^Sent only for patients with sexual concerns referred for sexologist consultation.

^l^The experience questionnaire will be specific for each supportive care intervention.

^m^Focus groups with participants of each supportive care intervention.

### Eligibility Criteria

Patients are eligible if they have histological confirmation of cancer of any type and stage, are aged ≥18 years, and provided written informed consent. For the primary end point, all patients included in SC pathways (ie, who received a formal SC needs assessment and tailored multidisciplinary referrals) will be invited to participate. As for the secondary end points, any patient participating in SC interventions may be invited to participate. As for health care providers, those who assist the patients included in the SC pathways, care managers, and coordinators involved are eligible. If a patient withdraws consent for the study, no further study-specific evaluations will be performed, and no additional data will be collected.

### Integrated SC Pathways

At Gustave Roussy, integrated pathways of SC were co-designed with patients, providers (including nurses, oncologists, radiotherapists, surgeons, and SC specialists), sociologists, care managers, technology experts, and implementation scientists. These pathways aim to provide a formal SC needs assessment and tailored multidisciplinary care referral across the entire cancer care continuum, leveraging mechanisms that ensure the inclusion of patients considered vulnerable. During the co-design phase, each pathway carefully considered the available scientific evidence and the needs of patients and providers according to tumor type and disease stage. Shortly after the moment of diagnosis, the objective of the integrated SC pathways is to anticipate and prevent treatment-related burden. An autoevaluation of patient’s symptoms and SC needs alongside a health care provider’s assessment are conducted to implement a personalized SC plan. This pathway is being implemented for patients with brain, head and neck, thoracic, and endocrine tumors and will be adapted and expanded to other tumor types, including breast cancer (long-term fatigue after breast cancer prevention of cancer-related fatigue and cluster of long-term behavioral symptoms pathway). During treatment, the goal is to continuously monitor and manage the toxicity of systemic therapies at an earlier stage, reduce symptom burden, and avoid recourse to emergency care. A total of 2 pathways are implemented: a remote patient monitoring pathway using electronic weekly patient-reported outcomes (electronic patient-reported outcomes version of the common terminology criteria for adverse events) with nurse navigation [19] and an immunotoxicity management pathway with a multidisciplinary expert team providing mobile evaluation for hospitalized and ambulatory patients and follow-up consultations. Both the remote patient monitoring pathway and the immunotoxicity management pathway are agnostic (any cancer type and stage). After treatment, the objective is to mitigate long-term treatment-related toxicities, accelerate rehabilitation, give support during long adjuvant treatments, promote healthier behaviors, and facilitate social reintegration. This pathway is implemented for breast cancer [20] and encompasses a personalized survivorship care consultation for needs assessment and SC referrals, the delivery of a survivorship care plan document, invitation to attend face-to-face survivorship educational group seminars, access to a mobile app delivering personalized education and self-management advice, and decision aids for physicians focused on SC needs. This pathway will be adapted and expanded to other tumor types in our institution.

A pathway implementation committee oversees the co-design and deployment process of the SC pathways, including the need for staff training or specific recruitments before the pathway is implemented in routine care. If staff replacements are needed, proper training is provided to new team members.

### Study Objectives

#### Primary Objective

The primary objective of this study is to evaluate the impact of an integrated proactive SC pathway in patients’ distress and unmet needs at 12 weeks’ follow-up in difference phases of the cancer continuum (at diagnosis, during treatment, and survivorship phase). Distress and unmet needs will be measured by the National Comprehensive Cancer Network (NCCN) Distress Thermometer and Problem List, respectively [30,31]. For patients participating in the immunotoxicity management pathway, the impact of the pathway will be evaluated at 4-week follow-up.

#### Secondary Objectives

The study’s secondary objectives are to (1) evaluate the *Reach* of the pathway through the absolute number, proportion, and representativeness of patients who participated in the SC needs assessment and in each SC intervention; (2) evaluate the microlevel *Efficacy* of the pathway and each SC intervention through the impact of the pathway and each SC intervention on patients’ quality of life, symptom burden, and distress; (3) evaluate the *Adoption* of the pathway through the absolute number, proportion, and representativeness of physicians and patients engaged with the pathway and each SC intervention; (4) evaluate barriers and leverages for pathway *Implementation* through collection of patient, health care provider, and organization experience; and (5) plan for *Maintenance* of the pathway through a cost-consequences analysis, with analysis of health resources and costs related to hospital services and efficacy outcomes (quality-adjusted life years [QALYs]) over a 6-month follow-up.

### Study Assessments

The NCCN’s Distress Thermometer and Problem List [30] will be used to measure patients’ distress and unmet needs at baseline and after 12 weeks and 24 weeks. In addition, we will evaluate at 12 weeks the Reach as the absolute number, proportion, and representativeness of patients: offered to participate in a SC pathway, who accepted the participation in a SC pathway, and for whom each type of SC strategy was indicated.

At baseline, a socioeconomic questionnaire and a health literacy questionnaire [32] will be completed. Quality of life will be evaluated at baseline, 12 weeks, and 24 weeks with the EQ-5D-5L [33] in the overall cohort and the 30-item European Organization for Research and Treatment of Cancer Quality of Life Core Questionnaire [34] for patients with breast cancer participating in fatigue prevention pathway. Symptom burden will be evaluated at baseline, week 12, and week 24 with the MD Anderson Symptom Inventory [35] in the overall cohort and relevant questionnaires for specific patient cohorts, such as the Hospital Anxiety and Depression Scale [36] for patients in the frailty prevention pathway (breast cancer) and patients referred to SC services for emotional distress and anxiety, the Insomnia Severity Index [37] for patients referred for SC strategies for insomnia, and the 22-item European Organization for Research and Treatment of Cancer Sexual Health Questionnaire [38] for patients with sexual concerns referred for sexologist consultation. In addition, patients with brain, lung, head and neck, and neuroendocrine tumors participating in the pathway to prevent treatment-related burden will also complete a vulnerability questionnaire at baseline.

Adoption at week 12 and week 24 will include metrics focused on (1) physicians: absolute number, proportion, and representativeness of physicians referring patients to the SC pathways and (2) patients: absolute number, proportion, and of patients that fully adopted each SC intervention (eg, in the after cancer pathway: attendance to consultations, seminaries, classes, completion of a SC program, and use data). To evaluate implementation, we will look at patient, provider, and organizational experience (levers and obstacles to proper implementation) within the proactive SC pathways at week 12 of follow-up. A patient experience questionnaire, the Patient Assessment of Chronic Illness Care [39], will be applied at week 12. In addition, an ad hoc patient satisfaction questionnaire (5-point Likert scale) will also be collected at 12 weeks after pathway delivery. This questionnaire includes core common questions for all pathways (overall satisfaction, perceived usefulness of the needs assessment and supportive are services proposed, clarity of the recommendations, and ability to comply with the SC plan recommended) and specific satisfaction and perceived usefulness questions for SC services used in each pathway; a free text field for comments is also included. All these quantitative analyses will be enriched with preplanned qualitative analysis with focus groups with patients, providers, and implementation team to assess experience, satisfaction, and contextual factors that influence implementation [40]. Maintenance of the pathway will be performed through a cost-consequences analysis. Hospital costs from the perspective of the French national health insurance will be assessed during the study period. This will be calculated by administrative data review of internal allocated resources for pathway delivery and estimated costs of use of hospital services (unplanned hospitalization, consultations, and emergency visits recorded in the electronic medical records). QALYs will be measured using utility values derived from the EQ-5D-5L. QALYs will be computed combining survival time by utility values.

Assessments at week 4 will be conducted exclusively with patients participating in the immunotoxicity management pathway (Table 1).

### Statistical Considerations

The NCCN’s Distress Thermometer scores are based on mean and SD values at baseline and have been used in heterogeneous populations [31,41]. To estimate the expected effect of the integrated and proactive SC pathway on Distress Thermometer scores, a pilot evaluation will be conducted to calculate the formal sample size needed for efficacy evaluation. According to the sample size requirements for pilot studies proposed by Teare et al [42], pilot evaluations with a minimum of 70 patients will be performed to generate NCCN’s Distress Thermometers mean scores and SDs before and after going through a SC pathway (70 patients for each SC pathway).

The primary analysis will compare patient distress scores by the NCCN’s Distress Thermometer measured before and after the intervention (baseline and week 12) using the Wilcoxon signed rank test.

For secondary analyses, the scores and subscores of the patient-reported outcome measurements (EQ-5D-5L, MD Anderson Symptom Inventory, Hospital Anxiety and Depression Scale, Insomnia Severity Index, 30-item European Organization for Research and Treatment of Cancer Quality of Life Core Questionnaire, and the 22-item European Organization for Research and Treatment of Cancer Sexual Health Questionnaire) and patient-reported experience measurement (NCCN Distress Thermometer) questionnaires and scales will be calculated using mixed models to take into account the repeated measures and the initial value before intervention.

All patients receiving SC interventions and referrals as well as health care providers and care coordination professionals performing activities related to the SC clinical pathways (nurses, oncologists, gynecologists, surgeons, radiation oncologists, and members from the multidisciplinary SC team) may be included in the study. For the primary end point, any patient who received a needs assessment in the context of an integrated supportive pathway will be included. For the secondary end points, any patient participating in SC activities will be included.

For the cost-consequence analysis, costs from the perspective of the French national health insurance will be assessed during the study period. This will be calculated by administrative data review of internal allocated resources for pathway delivery and estimated costs of use of hospital services (unplanned hospitalization, consultations, and emergency visits recorded in the electronic medical records). Utility values will be assessed using the EQ-5D-5L. QALYs will be computed combining survival time by utility values. Long-term efficacy will be evaluated with the same efficacy outcomes at 6-month follow-up.

All analyses will be performed using SAS (version 9.4; SAS Institute) and R (version 4.0.3). Statistical significance will be defined with a *P*<.05.

### Data Collection, Management, and Auditing

The Biostatistics and Epidemiology unit at Gustave Roussy implemented an electronic case report form (eCRF) to allow secure online-direct data collection using REDCap. Each user has personal identifiers (user ID and password), and data access is strictly limited according to profiles: (1) hospital clinical research assistant (CRA), allowing data entry on the eCRF; (2) data manager, allowing the first data monitoring, perform consistency checks, and edit requests for clarification addressed to the investigator or hospital CRA; and (3) investigator profile, enabled to sign and validate the data electronically. Electronic learning is mandatory to access the eCRF. The password is configured when the profile is activated and must be changed every 6 months. For each patient included, the eCRF has to be completed by hospital CRA and signed by a study investigator. An audit trail within the system tracks all the changes made to the data. Data sources of the study include the eCRF, data from the WeShare platform, and data from the technology providers of remote care interventions (Resilience Care). SC referrals and attendance will be captured in the eCRF of the study. Attendance to each of the SC strategies will be retrieved from the electronic medical records or from a specific attendance-log database in the case of integrative therapies. Usability data from technology-enabled SC interventions will also be retrieved and transferred by Resilience Care. Patient-reported outcomes data will be collected and hosted by the WeShare platform and transferred to the investigator at the end of the study. Both patient-reported outcomes data and usability data from Resilience Care are interoperable with Redcap. Data collected will be managed in the Biostatistics and Epidemiology unit at Gustave Roussy. Standard institutional practices will be followed to maintain the confidentiality and security of data collected in this study. A copy of the consent form and documentation of consent will be stored in a locked cabinet or an encrypted, password-protected computer drive. All protected health information collected from the study eCRF will be encrypted and password protected. If any questionnaire is filled out on paper, this will be stored in a locked cabinet in a secured office in Gustave Roussy. Data will be stored until data analysis is complete, and then the data will be transferred to a centralized repository. Access to the repository will be limited to the principal investigator, coinvestigators, and associates from the original study team. Future studies requesting the use of the data must either be related to the original research study or will require separate institutional review board approval. To guarantee the authenticity and the credibility of the data in conformity with good clinical practices, auditing and quality assurance systems include (1) study management in accordance with standardized procedures at Gustave Roussy and (2) quality control performed by the CRA. Particularly, it is the responsibility of the CRA to (1) check that the investigator’s file is correctly and regularly updated; (2) verify the signatures and validity of consent forms, fulfillment of eligibility criteria, validity of evaluation criteria, and adverse events; and (3) assure that reporting requirements are met. Regular meetings, held at least monthly, with the investigator team and study coordinator ensure a thorough review of study procedures, provide periodic updates on study progress (including patient enrollment numbers vs expected numbers), and address any procedural issues.

### Equity, Diversity, and Inclusion

To ensure equity, diversity, and inclusion, social determinants of health and health-related social risks are screened at pathway entry. In addition, SC services targeting actionable unfavorable social determinants of health, such as social services and financial counseling, referrals to smoking and alcohol cessation programs, and nutritional counseling, are part of the actions delivered in the context of the pathways. In addition, plain language standards are applied to educational materials used to communicate pathway activities toward patients. An objective guidance on equity, diversity, and inclusion is publicly available for researchers and providers involved in this study via the WeShare platform [28], and a cultural competency training is being developed and will be recommended for all providers involved in pathway activities (screening and care delivery). Social determinants of health and demographics are collected during the study and will allow to monitor the participation of populations that have been historically excluded in SC interventions.

### Patient Involvement in the Study

The SC pathways implemented in this study are co-designed with patient representatives upfront before deployment. Particularly, the study team meets with patient representatives at four key moments to foster cocreation: (1) at conceptualization to identify patient’s SC needs, (2) during development of pathway components giving input and ideas of components to prioritize and how to model them, (3) just before implementation, and (4) after implementation. In addition, a patient representative is a member of the operational team (JA). The study protocol was also discussed in detail and approved by patient representatives. Across the study, qualitative and quantitative assessments of patient experience are planned. These data will serve to continuously improve pathway components and delivery to meet the needs of the patients.

### Ethical Considerations

This study received regulatory approval by the institutional review board of Gustave Roussy and by the French national review board on September 4, 2023 (ID-RCB 2023-A01225-40). All participants (patients, providers, and managers) will provide informed and signed consent before taking part in the data collection process. Information that could potentially identify participants will be securely stored in a password-protected, locked database. Whenever possible, data will be deidentified through codification. Participants will not receive any monetary compensation for taking part in the study.

## Results

As of February 2024, the evaluation of the SC pathway to prevent treatment-related burden for patients considered vulnerable with brain, head and neck,

thoracic, and neuroendocrine tumors has started, and 12 patients were enrolled in the study. The evaluation of the SC pathway for preventing treatment-related burden after breast cancer and the proactive survivorship care pathway (breast cancer) is expected to start in April 2024. Data will be analyzed once at least 70 patients have completed the study evaluations for one of the pathways. Results of this study will be sent for publication in peer-reviewed journals and will also be presented to the multidisciplinary implementation team so that SC pathways can be refined accordingly to better serve the needs of patients and providers. The clinical trial is registered at ClinicalTrials.gov (NCT06479057).

## Discussion

### Principal Findings

The proposed study will evaluate the implementation of distinct integrated proactive pathways of SC that are offered to patients treated at Gustave Roussy. Our study has several strengths. It is a master protocol for a prospective study on patients diagnosed with any type of cancer, across different moments of the cancer care continuum, using the RE-AIM framework to guide its evaluation. Quantitative analyses will use a variety of data, including validated patient-reported outcomes measures; experience and satisfaction surveys; and administrative, sociodemographic, health literacy, and clinical data. Furthermore, these will be enriched with preplanned qualitative assessments (focus groups) with several stakeholders. This study design will allow for analysis of patterns of unmet needs that are currently difficult to generalize. In addition, it may serve as an umbrella protocol for ancillary prospective studies focusing on each SC intervention. It is expected to lead to a higher level of coordination than the one achieved by independently conducted studies.

Despite the existing literature on interventions addressing SC needs in patients with cancer, their implementation has been suboptimal due to inconsistency of empirical evidence for interventions and the difficulties experienced by policy makers and health service providers in finding and assessing the evidence for interventions [15,43]. Typical obstacles include low level of awareness among oncologist on SC services, lack of a comprehensive multidisciplinary SC team, fragmented and unclear communication and referral processes for SC interventions, nonstandardized delivery of needs assessment and tailored referrals, uneven access to SC services among patients considered vulnerable and minority groups, incorporation of nonevidence-based practices, reimbursement and care valorization issues, and lack of integration with community-based resources [7].

### Conclusions and Impact

This study will provide evidence on the implementation process of integrated, proactive SC pathways and will also inform future care delivery strategies to improve the reach and adoption of SC in oncology centers. It may serve as a model for other cancer centers trying to implement such integrated pathways. The analysis of quantitative and qualitative data collected from the patient, providers, and the health care organization for this study will be used to improve the design and delivery of SC pathways so that they meet the needs of patients and are feasible to be implemented according to a provider point of view. In addition, it will provide a detailed description of patients’ unmet SC needs across the cancer care continuum in multiple cancer types.

This study will also add evidence on using technology to facilitate the delivery and evaluation of SC pathways. More specifically, for care, electronic patient-reported outcomes are integrated with medical records and used for needs assessment to screen patient vulnerability status being used to perform tailored SC referrals since diagnosis. Technology is also used for remote patient monitoring where electronic patient-reported outcomes are transmitted in real time to nurse navigators during active treatment phase with chemotherapy and targeted therapies. Several digitally enabled SC strategies are also implemented, such as physical activity, meditation, yoga, and cognitive behavioral therapies. Finally, for research purposes, technology is used to collect patient-generated data and clinical data for outcomes assessment.

This study has the potential to impact policy and global SC practices in oncology through several mechanisms. First, it can serve as a model to other cancer centers to build and implement coordinated and integrated SC pathways. It will report in detail the infrastructure required in cancer centers to deliver integrated SC. It will also introduce innovative methods to facilitate implementation, such as using technology to screen patients’ needs, monitor symptoms, deliver specific SC interventions, and partner with community-based SC infrastructures. Furthermore, the proactive supportive care pathway approach is innovative as it allows to anticipate SC needs in a tentative manner to prevent treatment-related burden instead of using the standard reactive supportive care pathway approach currently used in most institutions. Finally, the presentation of this study in medical conferences and the publication of its results both in peer-reviewed journals and in lay media channels will also contribute to raise awareness among cancer care providers and policy makers to stimulate care and research initiatives of innovative models of SC delivery to improve patients’ quality of life, addressing the physical and psychosocial needs of patients while improving the organization of health services.

## Abbreviations

CRA: clinical research assistant
eCRF: electronic case report form
NCCN: National Comprehensive Cancer Network
QALY: quality-adjusted life years
RE-AIM: Reach, Effectiveness, Adoption, Implementation, and Maintenance
SC: supportive care

## Data Availability

The data sets generated during and/or analyzed during this study will be available in the WeShare Data repository [28].
